# Exosome-like Nanoparticles, High in Trans-δ-Viniferin Derivatives, Produced from Grape Cell Cultures: Preparation, Characterization, and Anticancer Properties

**DOI:** 10.3390/biomedicines12092142

**Published:** 2024-09-20

**Authors:** Yury Shkryl, Zhargalma Tsydeneshieva, Ekaterina Menchinskaya, Tatiana Rusapetova, Olga Grishchenko, Anastasia Mironova, Dmitry Bulgakov, Tatiana Gorpenchenko, Vitaly Kazarin, Galina Tchernoded, Victor Bulgakov, Dmitry Aminin, Yulia Yugay

**Affiliations:** 1 Federal Scientific Center of the East Asia Terrestrial Biodiversity, Far Eastern Branch of the Russian Academy of Sciences, 690022 Vladivostok, Russia; yn80@mail.ru (Y.S.); zargalma2509@gmail.com (Z.T.); avramenko.dvo@gmail.com (T.R.); crab_ol@mail.ru (O.G.); mistletoe8@gmail.com (A.M.); bulgakov-dv@mail.ru (D.B.); gorpenchenko@biosoil.ru (T.G.); kazarin@biosoil.ru (V.K.); tchernoded@biosoil.ru (G.T.); bulgakov@ibss.dvo.ru (V.B.); 2 Advance Engineering School “Institute of Biotechnology, Bioengineering and Food Systems”, Far Eastern Federal University, 690922 Vladivostok, Russia; 3 G.B. Elyakov Pacific Institute of Bioorganic Chemistry, Far Eastern Branch of the Russian Academy of Sciences, 690022 Vladivostok, Russia; ekaterinamenchinskaya@gmail.com (E.M.); d_aminin@hotmail.com (D.A.)

**Keywords:** extracellular vesicles, cancer, callus culture, salicylic acid, *Vitis vinifera*

## Abstract

**Background**: Recent interest in plant-derived exosome-like nanoparticles (ENs) has surged due to their therapeutic potential, which includes antioxidant, anti-inflammatory, and anticancer activities. These properties are attributed to their cargo of bioactive metabolites and other endogenous molecules. However, the properties of ENs isolated from plant cell cultures remain less explored. **Methods**: In this investigation, grape callus-derived ENs (GCENs) were isolated using differential ultracentrifugation techniques. Structural analysis through electron microscopy, nanoparticle tracking analysis, and western blotting confirmed that GCENs qualify as exosome-like nanovesicles. **Results**: These GCENs contained significant amounts of microRNAs and proteins characteristic of plant-derived ENs, as well as trans-δ-viniferin, a notable stilbenoid known for its health-promoting properties. Functional assays revealed that the GCENs reduced the viability of the triple-negative breast cancer cell line MDA-MB-231 in a dose-dependent manner. Moreover, the GCENs exhibited negligible effects on the viability of normal human embryonic kidney (HEK) 293 cells, indicating selective cytotoxicity. Notably, treatment with these GCENs led to cell cycle arrest in the G1 phase and triggered apoptosis in the MDA-MB-231 cell line. **Conclusions**: Overall, this study underscores the potential of grape callus-derived nanovectors as natural carriers of stilbenoids and proposes their application as a novel and effective approach in the management of cancer.

## 1. Introduction

Exosomes, or exosome-like nanoparticles (ENs), are nano-sized extracellular vesicles secreted by various cell types and have gained significant attention due to their pivotal roles in cell-to-cell communication, disease progression, and more recently, as potential biomarkers [1]. In plants, while much is yet to be explored, these vesicles are believed to mediate various physiological processes, including stress responses and symbiotic interactions [2]. Plant ENs, much like mammalian exosomes, play pivotal roles in the transportation of diverse biomolecules and mediate intercellular, interspecies, and cross-kingdom communication [3,4,5]. They are integral in orchestrating developmental processes, bolstering the immune response, and mounting protective reactions to environmental stressors [6,7]. Broadly, the molecular composition of plant ENs mirrors that of their mammalian counterparts, with a spectrum of microRNAs (miRNAs), proteins, and secondary metabolites [8]. However, a distinctive feature of plant-derived ENs is their non-immunogenic nature, and their production on a large scale is more cost-effective [9]. A notable clinical application is the use of edible plant exosomes, specifically grape extracts, to prevent oral mucositis associated with chemoradiation treatment for head and neck cancer. This particular therapeutic approach completed its Phase 1 trial in 2022 (registered as Clinical Trial NCT01668849).

Grapevines (*Vitis* spp.) are particularly intriguing subjects for exosome research due to their significant economic and agricultural value, as well as their rich content of bioactive compounds. These compounds, especially resveratrol and its oligomers (e.g., δ-viniferin, ε-viniferin, pallidol, and others) found in grape seeds, skins, and leaves, have been extensively studied for their potential therapeutic benefits [10]. In particular, viniferins, including trans-δ-viniferin, exhibit antiproliferative effects on various cancer cell lines, indicating a potential role in inhibiting tumor growth [11,12,13,14]. Beyond its anticancer properties, trans-δ-viniferin also exhibits antibacterial, antiviral, and antioxidant properties [15,16,17]. One promising method for the targeted delivery of stilbenes and other components of grape extracts is their encapsulation in solid lipid nanoparticles (SLNs) [18]. However, SLNs face issues such as limited drug loading capacity, potential for drug expulsion, and stability challenges over time, which can affect their efficacy as drug carriers [19,20,21,22]. At the same time, plant ENs offer a natural and potentially safer alternative to SLNs, providing inherent bioactivity and biocompatibility. 

Grape-derived ENs (GENs) have attracted significant attention for their unique properties and promising therapeutic applications [23]. Ju et al. [24] isolated GENs from grape juice, highlighting their distinctive transport properties and biological functions. Notably, oral administration of grape exosome-like nanoparticles (GENs) provided protection to mice against dextran sulphate sodium-induced colitis [24], highlighting the potential of edible plant-derived ENs as nanotherapeutic agents or alternative drug delivery systems. This property was further demonstrated in a study where GENs efficiently delivered drugs such as metformin, doxorubicin, and tamoxifen to breast tumor cells [25]. The nanovesicles exhibited rapid cellular internalization and showed a significant tumor-killing capacity by inducing apoptosis and necrosis, underscoring their potential to enhance cancer therapy efficacy while reducing off-target effects. Di Raimo et al. [26] investigated the antioxidant properties of Exocomplex^®^, a mixture of ENs derived from various fruits and vegetables, including *V. vinifera*, in restoring redox balance in hydrogen peroxide-treated mice. Their findings demonstrated that Exocomplex^®^, rich in bioactive compounds, effectively restored redox balance, suggesting its potential for managing oxidative stress-related conditions. It has been shown that GENs comprise proteins, lipids, and microRNAs and are assimilated by intestinal macrophages and stem cells, playing a pivotal role in mediating interspecies communication [27]. Their influence extends to inducing the expression of genes imperative for maintaining intestinal equilibrium, such as anti-inflammatory cytokines, and antioxidation processes [27]. Further studies have shown the presence of ENs, ranging from 30 to 200 nm, in grape juice. From these GENs, 55 distinct proteins have been identified, and their presence has been confirmed across various grape tissues [28]. Anusree et al. [29] optimized the isolation process for GENs by refining filtration and centrifugation steps to handle the high content of sugars, fibers, and fats present in plant extracts. Intriguingly, it was determined that several proteins from GENs, such as the heat shock protein HSP70 and aquaporin, have an overlap with proteins commonly found in mammalian exosomes. Additionally, the enrichment of specific proteins within plant ENs serves as marker signals for this biological process. These proteins include tetraspanin 8 (TET8), secretory syntaxin penetration 1 PEN1, and the aforementioned HSP70 [8].

Plant cell cultures offer an innovative and sustainable approach to producing ENs, presenting numerous advantages for research and potential therapeutic applications. Firstly, plant cell cultures can be maintained in controlled environments, ensuring consistency in EN production. This controlled setting reduces the variabilities seen in whole plants due to external factors such as seasonal changes and pests [30,31,32]. Moreover, specific plant species or cell lines can be selected for targeted EN production based on desired traits or therapeutic potential [32,33]. Furthermore, plant cell cultures are scalable, allowing for mass production of ENs, catering to both research needs and potential industrial applications [34,35]. The absence of pathogens that can infect humans makes plant-derived ENs a safer alternative to those from mammalian sources, presenting fewer biocontamination risks [9,36,37,38]. With the growing interest in ENs as drug delivery vehicles and their inherent therapeutic properties, plant cell cultures could emerge as a pivotal production platform in the future [38,39]. However, exosome isolation from plant cell cultures or their corresponding culture media remains relatively unexplored, with just a handful of studies achieving success in this domain [40]. Notably, ENs have been successfully extracted from the cell cultures of various plant species, including *Aster yomena* [31], *Nicotiana tabacum* [30,41], *Craterostigma plantagineum* [30], *Panax ginseng* [32], *Salvia dominica* [42], *S. sclarea* [43], and *Arabidopsis thaliana* [44]. For example, studies on *A. thaliana* and *N. tabacum* cell cultures revealed that their ENs contain proteins associated with key biological processes, such as cell wall biogenesis and defense mechanisms, and microRNAs that are similar to those found in intact plants [30,44]. These findings highlight the ability of callus-derived ENs to reflect the physiological states of their parent plants. Additionally, ENs isolated from tobacco cell cultures have demonstrated the ability to enter both plant and mammalian cells, positioning them as a promising platform for producing nanovesicles for various biotechnological and therapeutic applications [41]. The hairy root cultures of *S. dominica* and *S. sclarea* can efficiently produce ENs that show potential in a cellular model of Parkinson’s disease, where they effectively entered cells and inhibited apoptosis [43]. Meanwhile, another study on ENs from *S. dominica* revealed strong pro-apoptotic activity in mammary and pancreatic cancer cells [42]. ENs from *P. ginseng* culture supernatants have been found to improve replicative senescence in human dermal fibroblasts and mitigate ultraviolet B radiation-induced pigmentation in human melanocytes [32]. Furthermore, *A. yomena* callus-derived ENs have demonstrated significant therapeutic potential in inflammatory diseases. These ENs were shown to inhibit immune responses and reduce symptoms in asthma models, showing therapeutic efficacy similar to that of dexamethasone [31]. Despite these promising discoveries, no studies to date have focused on the isolation or characterization of ENs from *Vitis* spp. (grape) cell cultures. Considering the potential of GENs, grape callus cultures could serve as a valuable model, not only for the biotechnological production of these nanovesicles, but also for advancing our understanding of their biogenesis and functional properties.

This study conducted a comparative analysis to characterize ENs derived from both grape calluses (GCENs) and berry juice (GENs). GCENs were found to mirror the morphology of those found in GENs and include similar miRNA and protein components. Additionally, GCENs were observed to accumulate significant amounts of trans-δ-viniferin derivatives. A cytotoxicity assay demonstrated that these GCENs decreased the viability of a triple-negative breast cancer cell line (MDA-MB-231) and markedly induced cell death through the activation of apoptosis and cell cycle arrest in the G1 phase.

## 2. Materials and Methods

### 2.1. Plant Material and Growth Conditions

The *Vitis vinifera* callus culture has been established and characterized previously [45]. The calluses were cultivated on solid Murashige and Skoog (MS) media supplemented with 0.5 mg/L of 6-benzylaminopurine and 2 mg/L of 1-naphthaleneacetic acid in 50 mL glass Erlenmeyer flasks and maintained at 25 °C in the dark. To induce a salicylic acid (SA) response, the calluses were cultivated in the presence of 200 µM concentration of the hormone for two weeks. Grapes (cv Cabernet Sauvignon) were purchased from a local market. 

### 2.2. Isolation of ENs

GENs were extracted from freshly pressed grape berry juice following a previously established protocol [44]. Cellular debris and other subcellular entities were removed by employing a series of centrifugation steps at 7000× *g*, 10,000× *g*, and 20,000× *g*, each lasting 30 min at 4 °C. The final supernatant was passed through a 0.45 μm nylon filter. This solution was ultracentrifuged at 100,000× *g* for 1 h at 4 °C, utilizing an Optima MAX-XP ultracentrifuge (Beckman Coulter, Brea, CA, USA). 

For the isolation of GCENs from the *V. vinifera* calluses, we utilized cells freshly collected from cultures aged for two weeks. These cells were rinsed thrice with potassium phosphate-buffered saline (PBS) at pH 7.4 to cleanse any remaining nutrient media. After the rinsing process, excess fluid was removed using a mesh filter, followed by cell homogenization using a blender. The procedures that followed were in line with the methods adopted for plant samples. EN residues derived from both the grape juice fluid and the calluses were subjected to two PBS washes and subsequently reconstituted in 1 mL of filter-sterilized PBS.

### 2.3. Scanning Electron Microscopy

The morphology of the purified ENs was characterized using scanning electron microscopy (SEM). Prior to analysis, the ENs were fixed in 4% glutaraldehyde for 20 min. The ENs were washed twice with PBS and spotted onto carbon-coated grids. The droplets were then air-dried. SEM images were obtained using a Merlin high-resolution microscope (Carl Zeiss, Jena, Germany) at an accelerating voltage of 2.0 kV.

### 2.4. Nanoparticle Tracking Analysis

The size distribution of the isolated ENs was measured as previously described [46] using nanoparticle tracking analysis (NTA) with a Nanosight NS500 instrument (Malvern Instruments, Malvern, UK), following the manufacturer’s protocol. Prior to NTA, samples were diluted in water to achieve an estimated count of approximately 10–20 particles per visual frame. A 60 s video capturing particle movement due to Brownian motion was recorded at 23 °C. The zeta potential of the samples was determined using the automated algorithm provided with the system. Video data, consisting of 10 recordings per sample, were analyzed using the NTA analysis software, version 2.2.

### 2.5. Isolation of RNA and miRNA, cDNA Synthesis, and PCR Analysis

The total RNA was extracted from both the plant and callus samples, followed by the synthesis of first-strand cDNA, according to previously described methods [47]. For miRNA isolation from the ENs, an initial treatment with 5 µg/mL Proteinase K (Sigma-Aldrich, Saint Louis, MO, USA) was performed for 10 min at 37 °C. This was followed by the addition of 5 mM phenylmethylsulfonyl fluoride (PMSF; Sigma-Aldrich) and a subsequent 10 min incubation at room temperature. To deactivate Proteinase K, the samples were heated to 90 °C for 5 min. The ENs were then treated with RNase A (final concentration 0.5 µg/µL, Thermo Fisher Scientific, Waltham, MA, USA) for 20 min at 37 °C. The “Lira” Kit (Biolabmix, Novosibirsk, Russia) was used for miRNA extraction, which included thorough on-column DNase digestion to eliminate potential DNA contamination. The concentration of the extracted miRNAs was determined using a specific microRNA assay kit (Thermo Fisher Scientific, Waltham, MA, USA) and measured with a Fluo-100 A fluorometer (Allsheng, Hangzhou, China).

For quantitative real-time PCR (qPCR), the HS-qPCR 2x SYBR Blue Master Mix (Biolabmix, Novosibirsk, Russia) was used on a CFX96 Real-Time System (Bio-Rad Laboratories, Hercules, CA, USA). Specific primers for *V. vinifera* genes, namely *TET8* and *PEN1* (refer to Supplementary Table S1), were selected, with the *EF* gene used as the reference gene for normalization.

In evaluating the miRNA species present within the GENs and GCENs, the stem-loop technique, as presented by Yang et al. [48], was employed. A standard stem-loop primer was incorporated during the reverse transcription process. For the qPCR, each miRNA-specific forward primer was paired with a universal reverse primer (details in Supplementary Table S1). 

Analysis comprised three distinct biological replicates from separate RNA or miRNA extractions. Additionally, for every biological replicate, three technical replicates were examined. The CFX Manager Software (Version 1.5; Bio-Rad Laboratories) facilitated data processing.

### 2.6. Isolation and Analysis of GCEN Protein Cargo

#### 2.6.1. Protein Extraction Procedure

GCENs sourced from the calluses underwent a treatment process with Proteinase K, as described above. The total proteins were then extracted, as described in previous research [44], and separated via a 12.5% SDS-PAGE. After electrophoresis, protein bands were visualized using Coomassie staining. Gel sections containing the targeted proteins were carefully excised into smaller pieces. These gel slices then underwent an in-gel trypsin digestion procedure (using Trypsin V511; Promega, Madison, WI, USA) according to the manufacturer’s instructions. Post-digestion, the resultant peptides were desiccated using a Concentrator Plus (Eppendorf, Hamburg, Germany).

#### 2.6.2. Mass Spectrometry and Protein Identification

The peptide mass spectra capture was conducted via an Autoflex MALDI-TOF mass spectrometer (Bruker Daltonics, Bremen, Germany) equipped with a nitrogen laser in a positive reflector configuration. A standard RP 700-3500 Da method was activated for the full capture of the desired mass spectrum. Instrument operation was managed through FlexControl software (version 3.4), utilizing a nitrogen laser’s automatic function. Spectra calibration was externally executed using the Protein Calibration Standard I along with other standard methods. Thereafter, the flexAnalysis software (version 3.4) was employed for peak extraction. The SNAP program helped in pinpointing the primary monoisotopic signals in the data. Dedicated methods were set for MS and MS/MS analysis. Each spectral representation was an average of 1500–5000 laser shots, pulled at the lowest laser power setting. Analysis of this data was carried out using BioTools software (version 3.2). For database mapping, the Mascot search engine was employed. Parameters set for this matching included a peptide and fragment mass tolerance of 0.5 Da and a minimum score of 40 for genuine identification.

#### 2.6.3. Western Blot Analysis

Proteins extracted from GENs and GCENs were also subjected to western blotting, employing rabbit polyclonal antibodies specifically against TET8 and HSP70 (PHY1490S and PHY0167, respectively; PhytoAB, San Jose, CA, USA). Bands that reacted to these antibodies were identified using anti-rabbit goat alkaline phosphatase secondary antibodies (ab97048, Abcam, Cambridge, UK) followed by CDP-Star™ Substrate (T2146, Thermo Fisher Scientific, Waltham, MA, USA) reagent, and visualized using a VersaDoc MP molecular imager system (Bio-Rad Laboratories).

### 2.7. Analysis of Secondary Compounds

#### 2.7.1. Chemicals

An analytical standard of trans-ε-viniferin was obtained from PanReac AppliChem (GmbH, Darmstadt, Germany). Solvents, including methanol (MeOH), formic acid, and acetonitrile, used for HPLC were purchased from Sigma-Aldrich (Saint Louis, MO, USA). All extraction solutions and eluents were prepared with ultra-pure water (Millipore, Bedford, MA, USA). All solvents were of analytical grade.

#### 2.7.2. Extraction

After isolation, the GCENs were weighed and dried using a Concentrator Plus (Eppendorf, Hamburg, Germany) at 30 °C for 3 h. The dried sediments were then weighed and extracted with 100% MeOH at a ratio of 50 mg of sediment to 1 mL of MeOH. The samples were treated with ultrasound for 30 min at 40 °C. The mixture was vortexed vigorously and allowed to stand at 4 °C overnight before analysis.

#### 2.7.3. Analytical Chromatography and Mass Spectrometry

Reversed-phase high-performance liquid chromatography with a diode array detector and electrospray ionization mass spectrometry (RP-HPLC-UV-ESI-MS/MS^2^) was applied for polyphenol determination, as previously reported [45], with some modifications. A 1260 Infinity analytical HPLC system (Agilent Technologies, Santa Clara, CA, USA) interfaced with an ion trap mass spectrometer (the Bruker HCT Ultra PTM Discovery System (Bruker Daltonik GmbH, Bremen, Germany)) was used for the analysis. An analytical Zorbax C18 column (150 mm, 2.1-mm i.d., 3.5 μm particle size; Agilent Technologies, USA) was employed for separation at 40 °C. The mobile phase for gradient elution, with a flow rate of 0.2 mL/min, consisted of 0.1% aqueous formic acid (A) and acetonitrile (B). The gradient elution started from 0% B, increasing to 50% over 40 min, and up to 95% by 50 min. UV spectra were recorded with a diode array detector (DAD) in the range between 200 and 600 nm. Chromatograms for quantification were recorded at a wavelength of 320 nm. The MS experiments employed a negative ion mode for detection. The instrument parameters were as follows: *m*/*z* detection range of 100–1000, nitrogen (N2) as the drying gas at a flow rate of 8.0 L/min, nebulizer gas pressure set at 25 psi, and an ion source voltage of 3.8 kV, with the drying gas maintained at 325 °C. Tandem mass spectra were recorded using Auto-MS2 mode with smart fragmentation, where the collision energy was ramped. The fragmentation amplitude was configured at 1 V.

High-resolution MS analyses were performed using a Shimadzu LCMS-IT-TOF system (Shimadzu, Japan), which integrates a tandem ion trap and time-of-flight mass spectrometer. Spectral data were obtained under ESI conditions with both negative and positive ion detection modes, achieving a resolution of 12,000. The instrument settings included an *m*/*z* detection range of 100–1000, nitrogen (N2) as the drying gas at a pressure of 200 kPa, a nebulizer gas flow rate of 1.5 L/min, an ion source potential ranging from −3.8 to 4.5 kV, and an interface temperature of 200 °C.

### 2.8. In Vitro Experiments

#### 2.8.1. GCEN Uptake by MDA-MB-231 Cells

GCENs were labeled with PKH26 (Lumiprobe, Moscow, Russia) according to the supplier’s protocol. Briefly, exosomes collected after 100,000× *g* ultracentrifugation (15 min, 4 °C) were incubated with PKH26 for 5 min at room temperature. The reaction was stopped by adding fetal bovine serum. Labeled exosomes were washed with PBS by ultracentrifugation, and the pellets were resuspended in MEM medium and incubated with MDA-MB-231 cells for 4 h and 24 h at 37 °C [49]. Laser Confocal Imaging was performed using LSM 710 LIVE scanning confocal laser microscopes (Carl Zeiss, Jena, Germany) with an excitation wavelength of 543 nm and an emission filter of 570–670 nm for PKH26 dye. DAPI was excited at 405 nm and captured with a 450/50 nm bandpass filter. The confocal photos were processed using LSM 510 Release version 4.2 and ZEN 2011 software.

#### 2.8.2. Cytotoxicity Assays

The human triple-negative breast adenocarcinoma MDA-MB-231 cell line (HTB-26™) and the normal embryonic kidney HEK-293 cell line (CRL-1573™) were obtained from ATCC (Manassas, VA, USA). MDA-MB-231 and HEK-293 cells were seeded (7 × 10³ cells per well) in 96-well plates and placed in a CO₂ incubator for 24 h to allow for adhesion. Afterward, 20 µL of GCEN solutions at various concentrations were added, and the cells were incubated for an additional 24 h. The plates were incubated at 37 °C with 5% CO_2_ for 24 h. After 24 h of incubation of the cells with GCENs, the medium was replaced with 100 µL of fresh medium, and 10 µL of MTT solution (5 mg/mL) was added. The cells were then placed in a CO₂ incubator for 4 h. Subsequently, 100 µL of SDS-HCl solution (1 g SDS/10 mL d-H_2_O/17 µL 6 N HCl) was added to each well and incubated for 18 h. The absorbance of the converted formazan dye was measured using a Multiskan FC microplate photometer (Thermo Fisher Scientific, Waltham, MA, USA) at 570 nm.

#### 2.8.3. Caspase 3/7 Activity

The Muse^TM^ Caspase 3/7 kit (Millipore) was used according to the manufacturer’s instructions. MDA-MB-231 cells were seeded at a density of 3 × 10^4^ cells/mL in a 12-well plate, and 24 h after adhesion, GCENs were added at various concentrations for an additional 24 h. To 50 μL of cell suspension, 5 μL of Muse^TM^ Caspase-3/7 working solution was added. The cells were then left in a CO_2_ incubator for 30 min for staining. Then, 150 μL of Muse^TM^ Caspase 7-AAD working solution was added to each sample and left for 5 min at RT. The stained cells were then counted on a Muse^TM^ Cell Analyzer (Luminex, Austin, TX, USA). The results were processed using Muse 1.5 analytical software (Luminex, Austin, TX, USA).

#### 2.8.4. Annexin V-AF 488/PI Staining

Phosphatidylserine externalization was assessed using flow cytometry with annexin V-AF 488 and propidium iodide (PI) double staining. MDA-MB-231 cells were seeded in 12-well plates at a density of 3 × 10^4^ cells/mL. After 24 h of incubation, exosomes at various concentrations were added, and the plates were incubated for an additional 24 h. Cells were harvested using trypsin-EDTA solution, stained with an annexin V-AF 488/PI kit (Lumiprobe, Russia), and analyzed using a NovoCyte flow cytometer (Agilent, Santa Clara, CA, USA).

#### 2.8.5. Cell Cycle Analysis

To 50 µL of cell suspension, 5 µL of Muse™ Caspase-3/7 working solution was added. The cells were then placed in a CO₂ incubator for 30 min for staining. Afterward, 150 µL of Muse™ Caspase 7-AAD working solution was added to each sample and incubated for 5 min at room temperature. The stained cells were then counted using the Muse™ Cell Analyzer (Luminex, Austin, TX, USA). The results were processed using Muse 1.5 analytical software (Luminex, Austin, TX, USA).

### 2.9. Statistical Analysis 

All experiments were performed in triplicate. Data were subjected to statistical analysis using one-way ANOVA tests. Data are expressed as the mean ± SE, and *p* < 0.05 was regarded as statistically significant. 

## 3. Results and Discussion

### 3.1. Isolation and Characterization of ENs

**Figure 1:**
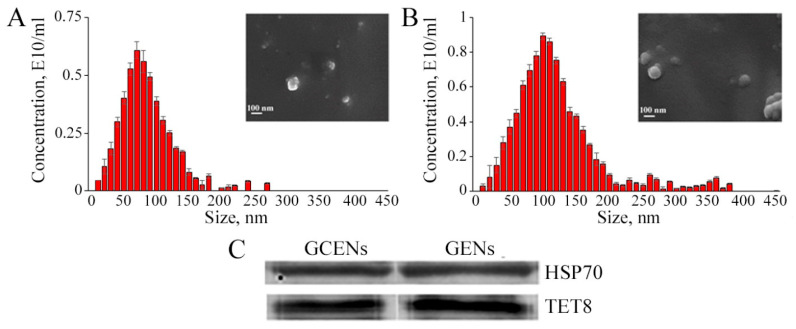
Scanning electron microscopy images and nanoparticle tracking analysis of GCENs (**A**) and GENs (**B**). The presence of HSP70 and TET8 proteins in ENs are shown in a western blot (**C**).

Exosome-like nanoparticles (ENs) were isolated from the grape calluses (termed GCENs) and berry juice (termed GENs) through ultracentrifugation. SEM analysis indicated that the size and structural integrity of the ENs remained stable across different samples. Notably, SEM images depicted the ENs’ diameters ranging from approximately 90 to 250 nm, accompanied by a slightly non-spherical contour (Figure 1A,B). Plant-derived ENs typically exhibit spherical, irregular, saucer-, or cup-shaped structures [50]. Such morphology likely depends on the type of originating cell, the isolation method, and the physiological conditions of the plant. Moreover, the distribution of embedded biomolecules within the lipid layer may play a role in shaping the membrane [51,52]. Intriguingly, nanoparticles obtained from grape berries showed a similar structural profile, with sizes ranging from 30 to 200 nm [28]. The existence of TET8 and HSP70 proteins was verified using western blotting prior to conducting the experiments (Figure 1C, Supplementary Figure S1), thus confirming the nature of isolated ENs. Importantly, it should be noted that HSP70 has also been previously detected in ENs derived from grape berry juice [28], emphasizing its relevance and recurrent association with *Vitis*-derived nanovesicles.

To discern the size distribution of the ENs from *V. vinifera*, NTA was employed. This revealed a diverse particle size range in the samples. The computed average dimensions for GCENs stood at 79 nm and for GENs at 100 nm (Figure 1A,B, and Table 1). This resemblance in size metrics mirrors the dimensions of ENs extracted from grapes [24,28] as well as other plant species [30,32,44]. Such consistency across different species hints at underlying regulatory frameworks steering EN formation. 

The zeta (Z) potential values, a measure of the surface charge, of the ENs were analyzed using NTA, and the findings are showcased in Table 1. The assessment of surface charges showed that both GCENs and GENs exhibited prominent negative Z potentials. Average measurements were recorded as −13.9 mV for GCENs and −30.3 mV for GENs. This negative surface charge of ENs suggests their capability to interact with positively charged entities, potentially enhancing specific cellular uptake or binding events. Previous Z potential measurements of GENs indicated a predominantly negative charge, with values ranging from −69.6 mV to +2.52 mV and an average potential of between −26.3 mV and −8.14 mV [53]. The negative surface charge of ENs is a key factor in their recognition and uptake by macrophages [54]. This charge is largely due to the presence of glycosyl inositol phospho ceramides, a type of sphingolipid, in the exosomal membranes [55]. The Z potential of plant exosomes is influenced by various factors, such as buffer concentration, presence of detergent, ionic strength, and pH, which determine their colloidal stability [56].

Using NTA, the concentration of ENs within the examined samples was further determined. The yield was calculated by gauging the vesicle concentration relative to 1 g of callus or plant matter. For *V. vinifera* calluses and berry juice, EN concentrations were measured at 1.2 × 10^10^ and 2.96 × 10^10^ particles per gram of fresh matter, respectively (Table 1). This indicates that the highest count of ENs was sourced from the grape juice. A previous study by these authors also showed slightly lower exosome contents in cultured *Arabidopsis* cells compared to apoplastic fluid from the same plant. However, in terms of absolute values, both values fall within the range of concentrations previously reported for various plant species [50]. For example, even lower content was noted in tobacco calluses, reaching 0.057 × 10^9^ per gram of fresh weight [41], while the yield from ginseng roots was 1.87 × 10^11^/g [57].

Plant-derived EN yield and concentration can be influenced by various factors. Viršilė et al. [58] found that these characteristics are specific to plant species, with *Artemisia absinthium* showing the highest yield. Lobb et al. [59] highlighted the impact of isolation methods, with centrifuge-based methods being more effective than pressure-driven ones. Ban et al. [60] demonstrated that low pH conditions can increase exosome yield, while Jang et al. [57] proposed a combination method of ultracentrifugation and ExoQuick precipitation for high-purity and high-stability exosome isolation from ginseng. 

### 3.2. Expression of Genes Involved in GCEN Biogenesis

**Figure 2:**
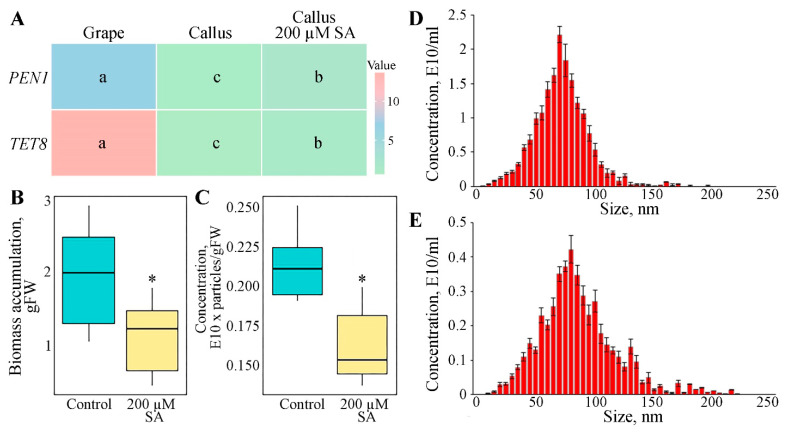
Expression levels of *VvTET8* and *VvPEN1* genes in grape berries and callus cultures (**A**). Biomass accumulation (**B**), and GCEN concentration (**C**) in grape calluses grown under control conditions and with 200 µM of salicylic acid (SA). Nanoparticle tracking analysis of GCENs isolated from control (**D**) and SA-treated calluses (**E**). Data shown are mean ± SE; n = 3. Different letters in the heatmap and asterisks denote significant differences at *p* < 0.05 (*) with Fisher’s LSD.

TETRASPANIN 8 (TET8) and PENETRATION 1 (PEN1) are pivotal proteins involved in the biogenesis and function of plant ENs, as they play crucial roles in the vesicular trafficking pathways that facilitate the formation and secretion of these vesicles [55,61]. The expression levels of *TET8* and *PEN1* genes in both callus cultures and grapes were analyzed using quantitative PCR and are depicted in the histogram (Figure 2A). For the *VvPEN1* gene, the expression level in the plant sample is markedly higher than in the callus culture, with the plant showing an approximately 4-fold greater expression. This suggests a greater involvement of *PEN1* in the biogenesis or function of exosomes within whole plant tissues compared to callus cultures. In contrast, the *VvTET8* gene exhibits an even more pronounced difference, with the expression in the plant being approximately 15 times higher than in the callus culture. Overall, these findings indicate that both *TET8* and *PEN1* genes are significantly more expressed in whole plants compared to *V. vinifera* callus cultures, highlighting their potential roles in the regulation of exosome biogenesis and functionality in different tissue types.

TET8, a tetraspanin protein, is a key component of the multivesicular body (MVB) pathway, which is essential for the sorting and packaging of cargo molecules into intraluminal vesicles that eventually form exosomes [8]. Tetraspanins are known to organize microdomains in cellular membranes, which are essential for the budding and release of exosomes [62]. Studies by Liu et al. [63] demonstrated that mutants lacking functional TET8 exhibit defects in exosome secretion, underscoring its importance in the biogenesis process. Interestingly, tetraspanins serve as markers for plant cells, enabling the distinction between self-nanovesicles and non-self-nanovesicles for pathogenic microorganisms [64]. Similarly, PEN1 (SYP121/SYR1), a vesicle-trafficking syntaxin protein, is implicated in the exocytic processes leading to the release of exosomes into the extracellular space [8]. It is involved in the SNARE (Soluble NSF-Attachment Protein Receptor) complex, which mediates the membrane fusion events necessary for vesicle trafficking [65]. PEN1 is also known for its role in plant defense mechanisms, particularly in the formation of cell wall appositions (papillae) at sites of pathogen attack [66]. The coordinated actions of TET8 and PEN1 are essential for the proper formation and secretion of plant exosomes, as well as for mediating intercellular communication and responses to environmental stimuli. Altered expression patterns of these key exosome biogenesis factors in grape callus cultures may potentially affect both the production of GCENs by cells and the loading of specific biomolecules into them.

### 3.3. Impact of Salicylic Acid on GCENs Biogenesis

Salicylic acid (SA) is known to be a key component in the plant defense response, acting as a signaling molecule to activate defense mechanisms [67]. Given the association between vesicle biogenesis and plant stress responses [2], we hypothesized that emulating such conditions could stimulate EN secretion in vitro. The callus cultures were treated with 200 µM SA to simulate stress conditions, and the impact on GCEN production and the expression of related genes was subsequently analyzed. The results revealed a significant upregulation in the expression levels of both *VvTET8* and *VvPEN1*, of 2.9-fold and 2.2-fold, respectively, following SA treatment, compared to the control (Figure 2A). These findings suggest that SA exerts a notable influence on the expression of key genes involved in the biogenesis of plant exosomes. Additionally, SA treatment led to a slight but statistically significant delay in grape cell biomass growth (Figure 2B). 

The observed upregulation of *VvTET8* and *VvPEN1* in response to SA suggests that SA may enhance the production of GCENs in grape callus culture. However, the GCEN content in SA-treated calluses was 1.3 times lower compared to untreated cells (Figure 2C). SA treatment did not significantly alter the size distribution profile of GCENs (Figure 2C). These findings highlight the complex interplay between SA-induced gene activation and EN formation in vitro. Understanding these mechanisms could have practical implications for manipulating vesicle-mediated processes in plant cells.

### 3.4. miRNA Composition of GCENs

**Figure 3:**
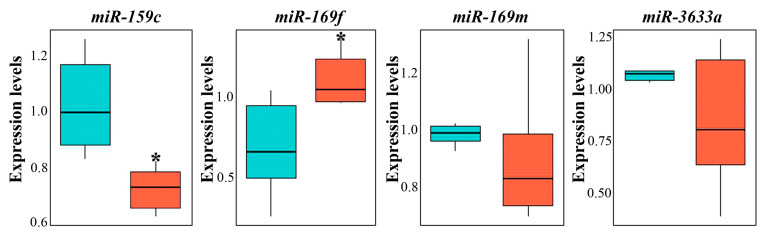
Expression levels of exosomal miRNA in GCENs and GENs. Data shown are mean ± SE; n = 3. Asterisks denote significant differences at *p* < 0.05 (*) with Student’s *t*-test.

The prevalence of small RNA patterns, including miRNAs and tiny RNAs within plant ENs, is well-documented [68,69,70]. In our study, we investigated a selection of miRNA species to ascertain their presence within GCENs compared to GENs. Representatives such as miR159c, miR169m, miR169f, and miR3633a, which have been previously identified as the most abundant species in the secreted fraction of ENs from the juice of grapes [24], were analyzed. Our results showed differential expression levels for these miRNAs. Specifically, miR159c exhibited expression levels 1.7 times higher in GCENs (Figure 3). In contrast, miR169f transcripts showed levels 1.5 times higher in GENs. Notably, miR169m and miR3633a displayed no significant differences in their expression levels between the different EN sources. Furthermore, the relative levels of individual miRNA variants within samples of both callus and berry ENs did not vary significantly, precluding any conclusions about the significant predominance of one isoform over the others. This consistency in expression levels suggests a stable representation of these miRNAs across different types of ENs derived from *V. vinifera*.

The presence of certain miRNAs in plant exosomes is crucial for intercellular communication under normal conditions and plays an important protective role when plants are attacked by pathogens [4,5]. Additionally, the potential therapeutic role of these miRNAs from consuming exosomes, either in purified form or directly from juice or plant parts, is also intriguing. For example, Xiao et al. [67] demonstrated that some plant miRNAs can target important human functional genes involved in inflammation, such as interleukins IL-2, IL-5, IL-6, and others, as well as the glucocorticoid-induced leucine zipper TSC22D3. miRNAs from watermelon exosomes may play a role in regulating various human cellular processes, including development, cytoskeletal dynamics, proliferation, and other essential functions [71]. Another study showed that maize miRNAs, which accumulate in the exosomes of this plant, are detected in the blood of pigs after consuming fresh maize [72]. At least one of the most abundant miRNAs in porcine blood and tissues, miR164a, can potentially influence functionally disparate target genes, such as chondroitin sulfate proteoglycan 4, homeobox protein OTX1, and pleomorphic adenoma gene-like 2. Teng et al. [23] reported that miRNAs found in grape exosome-like nanoparticles predominantly belong to the miR169 group and are similar to human hsa-miR-4480 and hsa-miR-4662a. However, whether this has any functional significance remains to be determined. 

### 3.5. Protein Composition of GCENs

The intrinsic ability of ENs to harbor a diverse range of proteins has been previously highlighted [69,70,73]. Proteomic analyses of GCENs confirm the presence of typical proteins, including vesicle-associated ones. The resultant profile underscored their crucial involvement in a multitude of cellular functions (Table 2, Supplementary Table S2). Among the identified proteins, several are intricately tied to signaling pathways, stress responses, cell wall organization, and enzymes pivotal for carbohydrate and lipid metabolism.

The presence of proteins such as heat shock proteins (HSPs), GDSL esterase/lipase, isoflavone reductase-like protein, and chitinases highlights ENs’ role in plant defense and stress responses. Moreover, the identified 70 kDa HSP is recognized as one of the marker proteins for plant-derived ENs [74,75]. In particular, HSP70 has been previously identified as a surface marker of ENs isolated from grapes, wheat, and Chinese ginseng [28,76,77], suggesting a potentially universal role in the formation or function of ENs across different plant species. GDSL esterase/lipase exhibits antimicrobial properties, providing a defense mechanism against pathogens. Isoflavone reductase-like protein is involved in the biosynthesis of flavonoids and isoflavonoids, compounds known for their roles in plant defense and stress tolerance. Chitinases break down chitin, a component of fungal cell walls. Annexins D1 and D2 have been recognized for their multifaceted roles in plants, influencing stress responses, development, and growth [78]. Because of their ability to bind and arrange membrane components these proteins also facilitate vesicle formation and transport in a calcium-dependent manner [79]. Proteins such as peroxidases, glutaredoxin-dependent peroxiredoxin, and universal stress protein PHOS34 are vital for maintaining redox homeostasis and enhancing tolerance to oxidative stress. These findings are consistent with numerous studies of exosomes isolated from various parts of different plant species, which have been shown to be rich in a variety of stress-related proteins (Table 2). This widespread presence of stress-related proteins in plant-derived exosomes suggests a conserved mechanism across different species for dealing with abiotic and biotic stresses, potentially highlighting the importance of ENs in plant resilience and adaptation.

The identification of structural enzymes like endoglucanase 1 and beta-galactosidase underscores ENs’ role in cell wall modification and remodeling. This observation aligns with previous findings where exosomes have been shown to carry components that are critical for cell wall dynamics [80,81,82]. Metabolic enzymes, including fructose-bisphosphate aldolase and phosphoenolpyruvate carboxylase, are crucial for glycolysis and carbon fixation, highlighting the involvement of ENs in essential metabolic pathways.

The data, together with the findings from previously published studies on the proteomic composition of exosomes from cultured plant cells [30,44], confirm that the proteins they contain mirror those found in whole plants. While the presence of enzymes involved in normal cellular functions can be attributed to the necessity of transporting these molecules into extracellular space, the presence of stress proteins is less straightforward to explain, particularly considering that the cultured cells are maintained under aseptic conditions. This observation suggests that even under controlled, sterile conditions, plant cells may still engage in the synthesis and export of stress-related proteins through exosomes, potentially as a preemptive or maintenance strategy to manage intracellular stress and maintain cellular homeostasis. 

**Table 2:** Protein composition of GCENs.

Protein	Functions	Occurrence in Plant-Derived ENs
Stress responses		
Heat shock 70 kDa protein	A molecular chaperone that plays a crucial role in maintaining cellular homeostasis and enhancing plant stress tolerance by assisting in the proper folding of newly synthesized proteins, preventing the aggregation of misfolded proteins, and aiding in the refolding or degradation of damaged proteins.	Citrus fruit juice [83], *Arabidopsis thaliana* leaf apoplastic fluid [2], juice from ginger roots [84], tomato fruits [85], broccoli roots [86], grape berries [24], *A. thaliana* cell culture [44], *Triticum aestivum* grass juice [76]
GDSL esterase/lipase	Participates in the metabolism of lipids by hydrolyzing ester bonds in fatty acids and contributes to plant health by modulating lipid signaling and maintaining membrane integrity.	*A. thaliana* leaf apoplastic fluid [2], sunflower extracellular fluid [87], juice from ginger roots [84]
Isoflavone reductase-like protein	Involved in the biosynthesis of isoflavonoids, which are important for plant defense mechanisms against pathogens and environmental stress.	Citrus fruit juice [83], *A. thaliana* leaf apoplastic fluid [2]
Abscisic stress-ripening protein 2	Plays a role in plant response to abscisic acid, which regulates stress responses and ripening processes.	*A. thaliana* leaf apoplastic fluid [2]
Class III chitinase	Breaks down chitin, a major component of fungal cell walls, providing a defense against fungal pathogens. Plays a morphogenetic role during apical growth, cell division, and differentiation.	*A. thaliana* leaf apoplastic fluid [2], sunflower extracellular fluid [87], tomato roots [88]
Class IV chitinase		Tomato roots [88]
Chitinase 5		Tomato roots [88]
Acidic endochitinase		Tomato roots [88]
Endochitinase EP3		Tomato roots [88]
Peroxidase	Catalyzes the breakdown of hydrogen peroxide, a reactive oxygen species, thus protecting cells from oxidative damage and playing roles in lignin formation and pathogen defense.	Citrus fruit juice [83]; sunflower extracellular fluid [87], grapefruit juice [89]
Peroxidase 4		Sunflower extracellular fluid [87]
Cationic peroxidase 1		Sunflower extracellular fluid [87], tomato roots [88]
Glutaredoxin-dependent peroxiredoxin	Reduces peroxides and protects cells from oxidative damage by using glutaredoxin as an electron donor.	-
Universal stress protein PHOS34	Involved in plant stress responses, helping to improve tolerance to various environmental stresses.	-
Retrotrans_gag domain-containing protein	Functions as part of retrotransposons, which can influence gene expression and genome stability.	-
Peptidyl-prolyl cis-trans isomerase	Catalyzes the isomerization of peptide bonds at proline residues, aiding in protein folding and function.	*A. thaliana* leaf apoplastic fluid [2], grape berries [24], citrus fruit juice [83]
5-methyltetrahydropteroyltriglutamate-homocysteine S-methyltransferase	Involved in the biosynthesis of methionine, which is crucial for protein synthesis and plays a significant role in the plant’s response to abiotic stresses.	*A. thaliana* leaf apoplastic fluid [2], olive vegetation water [90], tomato fruits [91], citrus fruit juice [83]
Usp domain-containing protein	Associated with stress responses, helping plants survive adverse conditions by stabilizing cellular proteins and membranes.	-
Annexin D1 and D2	Bind to phospholipids in a calcium-dependent manner, playing roles in membrane-related processes such as signal transduction and stress responses.	*A. thaliana* leaf apoplastic fluid [2], citrus fruit juice [83]
Cell wall remodeling and carbohydrate metabolism		
Endoglucanase 1	Breaks down cellulose into glucose units, aiding in cell wall remodeling and degradation.	Tomato roots [88]
Fructose-bisphosphate aldolase	Catalyzes a key step in glycolysis and gluconeogenesis, converting fructose-bisphosphate to glyceraldehyde-3-phosphate and dihydroxyacetone phosphate.	Grape berries [24], kiwi fruit pollen [92], fresh tea flowers [93], sunflower extracellular fluid [87]
Alpha-mannosidase	Involved in the modification and degradation of glycoproteins by cleaving mannose residues.	Sunflower extracellular fluid [87]
Putative polygalacturonase	Degrades pectin in the plant cell wall, facilitating cell wall modification during growth and development.	*A. thaliana* leaf apoplastic fluid [2], *Craterostigma plantagineum* cell suspension medium [27], grapefruit juice [85]
Beta-galactosidase	Hydrolyzes beta-galactosides into monosaccharides, involved in cell wall remodeling and carbohydrate metabolism.	*A. thaliana* leaf apoplastic fluids [2], *C. plantagineum* cell suspension medium [30], grapefruit juice [89], sunflower extracellular fluid [87]
Epidermis-specific secreted glycoprotein EP1	Plays a role in epidermal cell differentiation and defense against pathogens.	Tomato roots [88]
UDP-arabinopyranose mutase	Catalyzes the conversion of UDP-arabinopyranose to UDP-arabinofuranose, important for cell wall polysaccharide biosynthesis.	*C. plantagineum* cell suspension medium [30], citrus fruit juice [83], broccoli roots [86]
UTP-glucose-1-phosphate uridylyltransferase	Converts glucose-1-phosphate to UDP-glucose, a precursor for the synthesis of polysaccharides and glycoproteins.	Citrus fruit juice [83]
Primary and Secondary Metabolic Pathways		
Sucrose synthase 2	Catalyzes the reversible conversion of sucrose and UDP to UDP-glucose and fructose, playing a key role in sucrose metabolism and partitioning.	Citrus fruit juice [83], sunflower extracellular fluid [87], grapefruit juice [89]
Phosphoenolpyruvate carboxylase, housekeeping isozyme	Catalyzes the fixation of CO2 into oxaloacetate, a key step in photosynthesis and various metabolic pathways.	Citrus fruit juice [83], sunflower extracellular fluid [87], tomato fruits [91], grapefruit juice [89]
Phosphopyruvate hydratase (Enolase)	Catalyzes the conversion of 2-phosphoglycerate to phosphoenolpyruvate in glycolysis.	Citrus fruit juice [83]
Purple acid phosphatase	Hydrolyzes phosphate esters and anhydrides under acidic conditions, involved in phosphorus metabolism and mobilization.	Tea flowers [93], sunflower extracellular fluid [87]
Glyceraldehyde-3-phosphate dehydrogenase	Catalyzes a key step in glycolysis, converting glyceraldehyde-3-phosphate to 1,3-bisphosphoglycerate.	Olive vegetation water [90], citrus fruit juice [83], bitter melon juice [94], tea flowers [93]
Glyco_hydro_32C domain-containing protein	Likely involved in the hydrolysis of glycosidic bonds in complex carbohydrates.	-
Berberine bridge enzyme-like 2	Involved in the biosynthesis of alkaloids, which play roles in plant defense and secondary metabolism.	Olive vegetation water [90], grape berries [24]

### 3.6. Composition of Secondary Metabolites in GCENs

**Figure 4:**
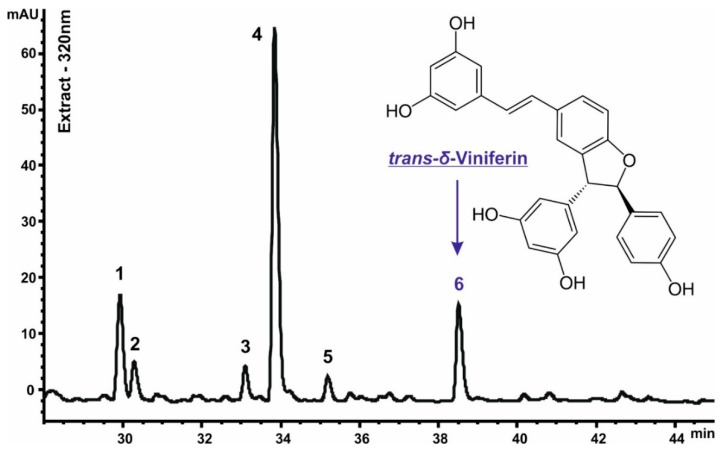
HPLC–UV (320 nm) profiling of stilbene derivatives from crude GCEN extracts, with peak numbers corresponding to the compounds listed in Table 3 and Supplementary Table S3. A schematic representation of the structure of trans-δ-viniferin (compound **6**) is also included.

The HPLC-UV-ESI-MS/MS2 method was utilized to determine the stilbene derivatives in the crude hydro-methanolic extracts of GCENs. High-resolution mass spectrometry confirmed compound identification. Six peaks (Figure 4) were identified as stilbenoids based on their chromatographic behavior, UV and mass-spectral data, and comparison with reference compounds and the scientific literature [95,96]. All identification data are detailed in Supplementary Table S2. The common dimeric stilbenoid trans-δ-viniferin (compound **6**), along with its monoglycosides (compounds **3** and **4**) and diglycosides (compounds **1** and **2**), were identified in GCENs. Additionally, compound **5** was recognized as the cis isomer of compound **4**, based on mass spectrometric similarity and UV spectra differences [95].

Table 3 presents the chemical composition of GCENs. Among the listed compounds, trans-δ-viniferin glycoside II is the most abundant, with a content of 480.4 ± 37.3 µg/g DW, significantly higher than the other compounds. Conversely, cis-δ-viniferin glycoside is the least abundant at 27.0 ± 2.2 µg/g DW. These findings suggest that glycosylated forms of viniferin are prevalent in the GCENs, potentially indicating their roles in plant defense mechanisms and their therapeutic applications. In the context of our findings, the presence of trans-δ-viniferin derivatives in GCENs underscores the potential of these exosomes in bioactive compound delivery. 

The increased δ-viniferin contents in exosomes could be attributed to several factors. Firstly, exosomes might preferentially encapsulate δ-viniferin due to its hydrophobic properties, aiding in its stabilization and transport [97]. Yet, glycoside derivatives are less hydrophobic and more water soluble than the corresponding aglycones. Additionally, the presence of specific transport proteins or vesicle packaging mechanisms within callus cells could facilitate the selective inclusion of δ-viniferin in exosomes. Further research is needed to elucidate the precise mechanisms governing δ-viniferin incorporation into exosomes.

Natural small molecular compounds from grapes, such as procyanidins and polyphenols, have been found in exosomes from berry juice [23]. Additionally, secondary metabolites in exosomes have also been identified in other plant species. For instance, ginseng exosomes contain ginsenosides, known for their anti-inflammatory and anticancer properties [98]. Citrus exosomes are rich in flavonoids such as naringin and hesperidin, which exhibit strong antioxidant activities [99,100]. Ginger exosomes have been shown to contain gingerol and shogaol, compounds with significant anti-inflammatory and anticancer effects [80,84,101]. ENs isolated from *Cannabis sativa* contained cannabidiol at levels dependent on the plant chemotype [102]. Exosomes isolated from *Solanum nigrum* berries were enriched with several chemical compounds that could define their anti-inflammatory activity in LPS-stimulated RAW264.7 cells [103]. These examples highlight the diverse and potent bioactivities of plant exosomal metabolites, further supporting the potential therapeutic applications of plant-derived ENs across various plant species.

### 3.7. Uptake of GCENs by Human Cells and Their Cytotoxic Effects

**Figure 5:**
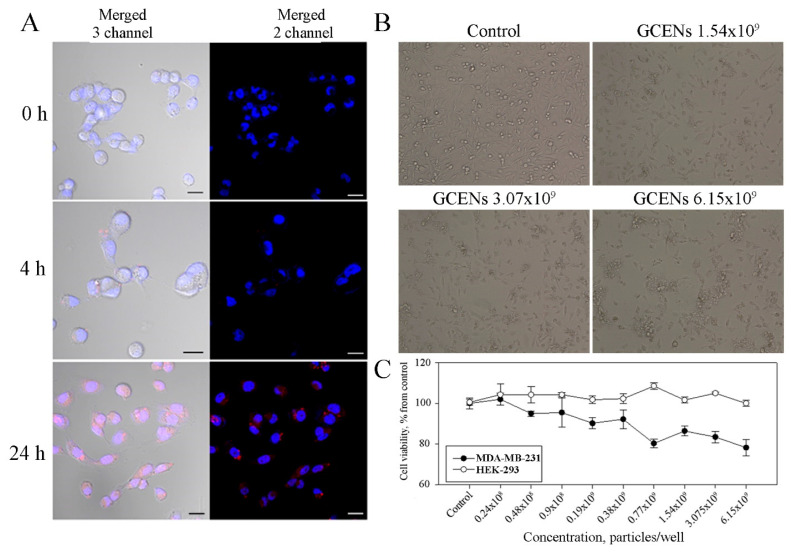
Confocal fluorescence imaging of GCENs labeled with PKH26, showing internalization in MDA-MB-231 cells after 4 and 24 h of incubation (**A**). The merged images display three channels: blue—DAPI stain for nuclei (excitation at 405 nm), red—PKH26 stain for exosomes (excitation at 543 nm), and transmitted light detection for a common view. Merged images from two channels show only the DAPI stain (blue) and PKH26 stain (red) for clarity. Light microscopy imaging of MDA-MB-231 cells illustrating cell morphology at different concentrations of GCENs (**B**), and the cytotoxic effect of GCENs on MDA-MB-231 and HEK-293 cells assessed by the MTT assay (**C**). Scale bar: 20 µm.

The internalization of plant ENs by target cells is crucial for effective drug delivery and therapeutic outcomes. The membranes of GCENs were labeled with the red fluorescent dye PKH26 to evaluate their ability to transfer into human cells. The model used in this study was the triple-negative breast cancer (TNBC) cell line MDA-MB-231, and fluorescence detection was performed using laser confocal microscopy (Figure 5). It was found that labeled GCENs rapidly entered the MDA-MB-231 cells, localizing in the perinuclear compartment, with approximately 50% of the cells exhibiting red fluorescence within just 4 h of incubation. After 24 h, a specific fluorescent signal was detected in over 90% of the TNBC cells treated with GCENs.

The literature shows that plant ENs can be internalized by mammalian cells through various mechanisms, such as endocytosis, plasma membrane fusion, phagocytosis, micro-, and macropinocytosis [33,89,104,105]. The uptake process is influenced by factors such as the size, charge, and surface properties of the ENs, as well as the type of target cell. Furthermore, the lipid composition of plant exosomes plays a crucial role in their uptake by mammalian cells. The presence of phosphatidic acid, a common lipid in plant exosomes, has been shown to enhance their fusion with mammalian cell membranes [106]. Importantly, the internalization of plant exosomes is not limited to in vitro studies but has also been observed in vivo after oral administration, providing evidence for their potential use in drug delivery systems [107].

Studies have shown that GENs can be internalized by various mammalian cells, including endothelial cells, lymphocytes, macrophages, and stem cells, through the endocytic pathway in an energy-dependent manner [23,24]. Given the morphological and molecular similarities between GCENs and GENs, it is highly likely that the GCENs in this study were also internalized into MDA-MB-231 cells via endocytosis.

Following confirmation that MDA-MB-231 cells internalize GCENs, the next step was to determine whether these exosomes cause cytotoxic effects. The results of the MTT assay demonstrate the impact of GCENs on the viability of HEK-293 cells (used as a control) and MDA-MB-231 triple-negative breast cancer cells (Figure 5). The data reveal that treatment with GCENs does not significantly impact the viability of HEK-293 cells across the tested concentrations, as cell viability remains consistently around 100% of the control. This indicates that the exosomes exhibit low cytotoxicity toward normal, non-cancerous cells. In contrast, the MDA-MB-231 cells show a gradual decrease in viability with increasing concentrations of exosomes. At the highest concentration tested (6.15 × 10^9^ particles/mL), cell viability dropped to approximately 70% of the control. 

Although it was not possible to establish a half-maximal inhibitory concentration (IC_50_) based on the MTT assay, the morphological analysis provided further insights into the impact of GCENs on MDA-MB-231 cells (Figure 5). In the control group, cells appeared healthy and proliferative, maintaining a typical spindle-like morphology. However, with the addition of GCENs at concentrations of 1.54 × 10^9^, 3.07 × 10^9^, and 6.15 × 10^9^ particles/mL, a clear dose-dependent reduction in cell density was observed. This decrease in cell number was accompanied by noticeable alterations in cell morphology, including cell shrinkage and loss of the characteristic spindle shape, which are indicative of stress or the onset of apoptosis. The obtained results might reflect the relatively moderate potency of the GCENs or the potential involvement of multiple pathways in mediating their effects. Nonetheless, the combined data from the MTT assay and morphological analysis strongly support the notion that GCENs can selectively affect cancer cell viability and morphology, making them a potential candidate for further exploration as an anticancer agent.

### 3.8. Apoptotic and Cytostatic Effects of GCENs

**Figure 6:**
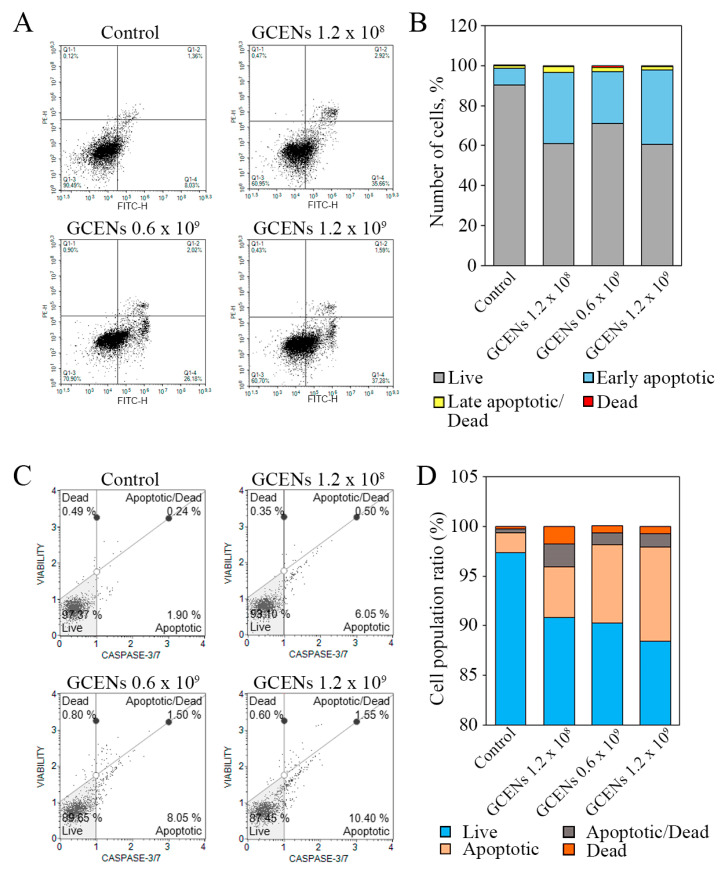
Analysis of cell death induced by GCENs in MDA-MB-231 cells. Flow cytometric analysis of apoptotic cell death using the fluorescent dye Annexin V-AF 488 (**A**,**B**) and a Caspase 3/7 activation assay (**C**,**D**) were performed after 24 h. Samples without EN treatment served as controls in each case.

The transition of phosphatidylserine (PS) from the inner to the outer monolayer of the plasma membrane is a hallmark of early apoptosis [108]. This inversion in MDA-MB-231 cells under the influence of GCENs was studied using flow cytometry and the fluorescent dye Annexin V-AF 488. The level of apoptotic cells was measured after 24 h of incubation with GCENs. The results showed that treatment with GCENs led to a significant decrease in the number of living cells (Figure 6A,B). The inversion of PS on the outer monolayer of the plasma membrane was observed after 24 h of exposure to GCENs. At a concentration of 1.2 × 10^9^ particles/mL, the number of early apoptotic cells significantly increased from 8.03% in the control to 37.28%. Additionally, there was an observed increase in cells at the late apoptosis/necrosis stage. The maximum effect was noted at a concentration of 1.2 × 10^8^ particles/mL, where the percentage of late apoptotic and dead cells rose to 2.92%, compared to 1.36% in the control cells.

Caspase-3 and caspase-7 are key executioner enzymes in the apoptosis process. They are activated in the later stages of apoptosis, leading to the cleavage of various substrates in the cell, ultimately causing cell death [109]. The apoptotic effects of GCEN treatments on MDA-MB-231 cell viability were assessed using Caspase 3/7 activation as an indicator of apoptosis. The viability profile was clearly impacted by GCEN treatment, with a notable shift in the proportion of live cells and an increase in apoptotic cells (Figure 6C,D). The data indicated a dose-dependent apoptotic effect of exosome treatment on cells. At the highest GCEN concentration (1.2 × 10⁹ particles/mL), the percentage of live cells significantly decreased to 88.45 ± 1.0%, compared to 97.33 ± 0.05% in the control group. Additionally, the number of apoptotic cells increased from 2.02 ± 0.12% in the control to 9.48 ± 0.92% following GCEN treatment. This trend was consistent, though slightly less pronounced, at lower concentrations of exosomes (Figure 6C,D). Our data indicate that the increase in apoptotic cell populations correlates with a decrease in cell viability, further demonstrating the pro-apoptotic potential of GCENs.

**Figure 7:**
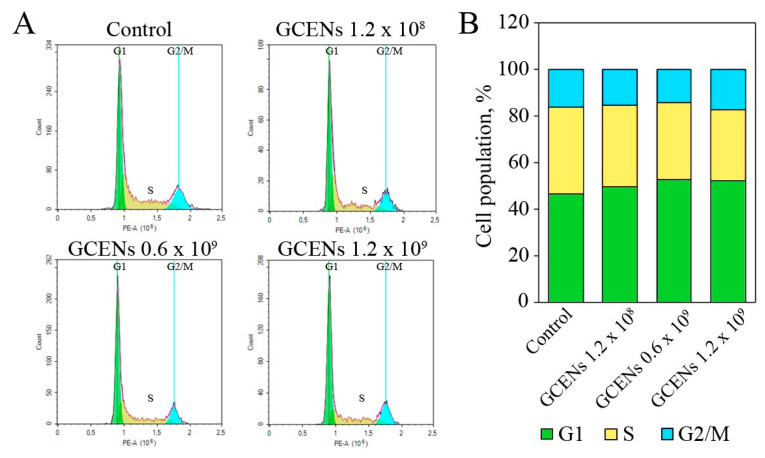
Flow cytometric analysis of MDA-MB-231 cell cycle arrest induced by GCEN treatment after 24 h (**A**), and quantitative results of cell cycle distribution (**B**). A sample without EN treatment served as the control. Green denotes the G1 phase, yellow denotes the S phase, and blue denotes the G2/M phase.

Flow cytometry was used to analyze the cell cycle distribution of MDA-MB-231 cells treated with GCENs (Figure 7A,B). The control cells predominantly remained in the G1 phase (45.95 ± 0.76%), with 37.04 ± 0.87% in the S phase and 15.97 ± 0.28% in the G2/M phase. Treatment with GCENs at 1.2 × 10^9^ particles/mL resulted in a significant increase in the G1 phase population (50.83 ± 0.04%) and a decrease in the S phase population (29.03 ± 2.03%), indicating a G1 phase arrest. Exosome treatment at 0.6 × 10^9^ particles/mL similarly caused an increase in the G1 phase population (52.54 ± 0.51%) and a reduction in the S phase population (32.71 ± 1.36%). At the lowest concentration of 1.2 × 10^8^ particles/mL, a slight increase in G1 phase cells (49.78 ± 1.57%) and a modest decrease in S phase cells (35.74 ± 0.4%) were observed. These data suggest that GCENs can induce cell cycle arrest in the G1 phase, which may contribute to their antiproliferative effects on cancer cells. The observed cell cycle disruption, along with the induction of apoptosis, underscores the potential of these exosomes as therapeutic agents in cancer treatment.

The ability of GCENs to induce apoptosis in cancer cells parallels the activity observed in ginger ENs, which have been shown to induce apoptosis, cell cycle arrest, and anti-metastatic effects in MDA-MB-231 cells [110]. These findings suggest a broader potential for plant-derived exosomes to modulate key cellular processes involved in cancer progression. GCENs also exhibit cytostatic effects by arresting the cell cycle in MDA-MB-231 cells. This activity is crucial for controlling the unchecked proliferation characteristic of cancer cells. Such an effect supports the hypothesis that plant-derived exosomes can interfere with cell cycle machinery, further corroborated by the work of Raimondo et al. [111], which demonstrated that lemon-derived nanovesicles inhibited the proliferation of various tumor cell lines. Interestingly, GENs have been reported to exert antitumor effects by increasing the expression of *Lgr5* and *BMI1* genes, which are markers of intestinal stem cells and regulators of stem cell growth and proliferation [27]. This highlights a unique mechanism by which plant-derived ENs may interact with mammalian cells to modulate gene expression, potentially affecting both cancer stem cells and normal stem cell niches.

Plant-derived ENs can carry various bioactive molecules, including proteins, lipids, miRNAs, and secondary metabolites, which can be transferred to mammalian cells. Some of these bioactive compounds are known to possess anticancer properties. Triple-negative breast cancer, (TNBC) relies heavily on the overactivation of the Wnt pathway [112]. Various stilbene derivatives, including the trans-δ-viniferin series, have shown inhibitory activity against the oncogenic Wnt pathway [113]. Therefore, the inhibitory effect of GCENs on MDA-MB-231 cells may be associated with the trans-δ-viniferin derivatives they contain. However, the antitumor activity of these compounds may also be exerted through other mechanisms. For instance, α-viniferin has been shown to induce apoptosis in non-small cell lung cancer by activating caspase-3 and cleaving poly(ADP-ribose) polymerase-1 (PARP-1) proteins, while also downregulating silent information regulator 1 (SIRT1), vimentin, and phosphorylated protein kinase B (AKT) proteins. Additionally, it facilitates the nuclear translocation of apoptosis-inducing factor (AIF), further contributing to the apoptotic process [114]. Another study demonstrated that both α-viniferin and ε-viniferin significantly inhibit epithelial–mesenchymal transition (EMT), a key process in cancer metastasis, and reduce invasion and migration in transforming growth factor beta 1 (TGF-β1)- or interleukin 1 beta (IL-1β)-induced non-small cell lung cancer cells [115]. Moreover, stilbenes have been reported to arrest the cell cycle in the G2/M phase in human hepatocellular carcinoma cells, increase reactive oxygen species (ROS) levels, enhance caspase-3 activity, and elevate the Bcl-2-associated X protein (Bax)/B-cell lymphoma 2 (Bcl-2) ratio, all of which suggest activation of the mitochondrial apoptotic pathway [116]. It is also known that trans-δ-viniferin can trigger mitochondrial membrane potential, leading to increased generation of ROS, which in turn induce apoptosis [14]. Additionally, the existing literature provides valuable insights into the potential in vivo efficacy of viniferin, a key component of GCENs. Studies have shown that viniferin can reduce cancer metastasis, inhibit tumor growth, and decrease tumor volume and mass in animal models [114,115].

Despite the promising findings, there are still many challenges to overcome, such as understanding the precise mechanisms of exosome uptake and their biodistribution in mammalian systems. Future research should focus on elucidating these mechanisms and exploring the therapeutic potential of plant exosomes for delivering various therapeutic molecules across species barriers.

## 4. Conclusions

In our study, we investigated the physical, chemical, and molecular biological properties of exosome-like nanoparticles isolated from grape calluses (termed GCENs) and berry juice (termed GENs). Our findings indicate that callus cultures generate ENs with dimensions and morphology closely resembling those extracted from berry juice. However, the concentration of ENs within calluses was lower than that observed in intact plant tissues, a feature associated with decreased transcriptional activity of factors such as *PEN1* and *TET8*. Additionally, the introduction of the plant stress hormone salicylic acid to the callus cultures significantly reduced EN production.

The detailed proteomic analysis of GCENs has revealed a diverse array of proteins involved in stress responses, cell wall remodeling, carbohydrate metabolism, and primary and secondary metabolic pathways. These vesicles also displayed a specific collection of EV-associated microRNAs that were similar to those found in the plant, though in reduced amounts. Notably, for the first time, grape callus-derived EVs were found to contain significant concentrations of stilbene derivatives, particularly trans-δ-viniferin isoforms.

GCENs demonstrated high efficiency in penetrating human tumor cells, as exemplified in the triple-negative breast cancer cell line MDA-MB-231. Moreover, they induced a dose-dependent apoptotic effect and cell cycle arrest in the G1 stage. Thus, grape callus cultures can serve as efficient, stable, and safe sources of ENs with significant biomedical potential.

## Figures and Tables

**Table 1:** Characteristics of ENs isolated from *V. vinifera* calluses and berry juice.

Samples	Average Size, nm	Ζ-Potential, mV	Concentration, Particles/g FW *
GCENs	78.8 ± 6.5	−13.9 ± 1.3	1.2 × 10^10^
GENs	99.7 ± 8.3	−30.3 ± 2.2	2.96 × 10^10^

* FW—fresh weight. Data shown are mean ± SE; n = 3.

**Table 3:** Chemical composition of GCENs (µg/g dry weight).

Compound	Trans-δ-Viniferin Diglycoside I	Trans-δ-Viniferin Diglycoside II	Trans-δ-Viniferin Glycoside I	Trans-δ-Viniferin Glycoside II	Cis-δ-Viniferin Glycoside	Trans-δ-Viniferin
No. of peak	1	2	3	4	5	6
Content	178.2 ± 36.4	61.4 ± 8.5	49.7 ± 3.5	480.4 ± 37.3	27.0 ± 2.2	136.7 ± 9.6

Data shown are mean ± SE; n = 3.

## Data Availability

The original contributions presented in the study are included in the article, further inquiries can be directed to the corresponding authors.

## Supplementary Materials

The following supporting information can be downloaded at: https://www.mdpi.com/article/10.3390/biomedicines12092142/s1, Table S1: List of primers used in this study; Table S2: Protein composition of ENs isolated from callus culture of *V. vinifera*; Table S3: List of phenolic compounds identified in *V. vinifera* callus-derived ENs by HPLC-UV-ESI-MS(MS^2^); Figure S1: Western blot analysis of ENs isolated from *V. vinifera* calli and berries juice. Proteins (40 µg) were resolved using a 10% SDS-PAGE gel. EVs marker proteins detected were: (**A**) HSP70 (71/72 kDa) and (**B**) TET8 (31 kDa).
