# Supplementary material for: Exosome-like Nanoparticles, High in Trans-δ-Viniferin Derivatives, Produced from Grape Cell Cultures: Preparation, Characterization, and Anticancer Properties
Source: Biomedicines. 2024 Sep 20;12(9):2142. doi: 10.3390/biomedicines12092142 (PMC11428831; doi:10.3390/biomedicines12092142)
Supplement: Supplementary file 1 [file biomedicines-12-02142-s001.zip › biomedicines-3200744-supplementary.pdf]

Exosome-like Nanoparticles, High in Trans-δ-Viniferin Derivatives, Produced from Grape Cell Cultures: Preparation, Characterization, and Anticancer Properties

Yury Shkryl [1], Zhargalma Tsydeneshieva [1,2], Ekaterina Menchinskaya [3], Tatiana Rusapetova [1], Olga Grishchenko [1], Anastasia Mironova [1], Dmitry Bulgakov [1], Tatiana Gorpenchenko [1], Vitaly Kazarin [1], Galina Tchernoded [1], Victor Bulgakov [1], Dmitry Aminin [3] and Yulia Yugay [1,*]

1 Federal Scientific Center of the East Asia Terrestrial Biodiversity, Far Eastern Branch of the Russian Academy of Sciences, 690022 Vladivostok, Russia; yn80@mail.ru (Y.S.); zargalma2509@gmail.com (Z.T.); avramenko.dvo@gmail.com (T.R.); crab_ol@mail.ru (O.G.); mistletoe8@gmail.com (A.M.); bulgakov-dv@mail.ru (D.B.); gorpenchenko@biosoil.ru (T.G.); kazarin@biosoil.ru (V.K.); tchernoded@biosoil.ru (G.T.); bulgakov@ibss.dvo.ru (V.B.)

2 Advance Engineering School “Institute of Biotechnology, Bioengineering and Food Systems”, Far Eastern Federal University, 690922 Vladivostok, Russia

3 G.B. Elyakov Pacific Institute of Bioorganic Chemistry, Far Eastern Branch of the Russian Academy of Sciences, 690022 Vladivostok, Russia; ekaterinamenchinskaya@gmail.com (E.M.); d_aminin@hotmail.com (D.A.)

* Correspondence: yuya1992@mail.ru; Tel.: +7-4232-312129; Fax: +7-4232-310193

Supplementary Table S1. List of primers used in this study

Gene (GenBank accession no.)	Forward (5′–3′)	Reverse (5′–3′)
EF1α (XM_002284888)	GAACTGGGTGCTTGATAGGC	ACCAAAATATCCGGAGTAAAAGA
TET8 (XM_002271648)	TGGCAAGGTGTGTCAGAGC	CTTCAGATTGTCCAGCACTC
PEN1 (XM_002263950)	CCTCAAGGAACTCCACCAG	TGGAGGTGGAGATGTGCTA
Universal stem-loop primer	GAAAGAAGGCGAGGAGCAGATCGAGGAAGAAGACGGAAGAATGTGCGTCTCGCCTTCTTTCNNNNNNNN	
Universal reverse primer	-	CGAGGAAGAAGAAGACGGAAGAAT
miR159c (NR_127742)	CTTGGATTGAAGGGAGCTC	-
miR169m (NR_127772)	ACTAGCCAAGGATGACTTGC	-
miR169f (NR_127772)	CCAGCCAAGGATGACTTGC	-
miR3633a (NR_127885)	ATAGGAATGGATGGTTAGGAG	-

Supplementary Table S2. Protein composition of ENs isolated from callus culture of *V. vinifera*

Name of protein	Score	Mass	Peptides	Unique peptides	Observed mass/sequences
Alpha-mannosidase	68	137475	10	10	902.5117 [K.SLEVIWR.G];<br>1144.6678 [R.MLSGYYLAAR.Q];<br>1284.6264 [K.THDYFPYADR.I];<br>1351.6711 [K.FVFAEMAFFSR.W];<br>1445.8373 [K.SLVVVAYNPLGWK.R];<br>1479.7296 [R.KFVFAEMAFFSR.W];<br>1601.9601 [K.SLVVVAYNPLGWKR.T];<br>1958.8648 [R.GNYYMSIDLLGDGAQWR.R];<br>1965.9885 [K.LNVHLVPHSHDDVGWLK.T];<br>1983.0003 [R.GGPVDNSTLVVELGPMEIR.T]
Putative polygalacturonase	32	52148	3	3	2716.4733<br>[K.AFQAAIDHLSQFASDGGSQLFVPPGR.W];<br>2716.5688<br>[K.AFQAAIDHLSQFASDGGSQLFVPPGR.W];<br>2716.7556<br>[K.AFQAAIDHLSQFASDGGSQLFVPPGR.W]
Beta-galactosidase	80	83065	1	1	1983.9714 [R.GSFINYYMYHGGTNFGR.T]
Class III chitinase	152	31839	5	5	1331.7860 [K.YSYVNIAFLNK.F];<br>1512.7266 [K.YYDDQSGYSSSIK.S];<br>3226.8035<br>[R.IFMGLPASSAAAGSGFIPANVLTSQILPVIKR.S];<br>3226.8482<br>[R.IFMGLPASSAAAGSGFIPANVLTSQILPVIKR.S];<br>3242.8219<br>[R.IFMGLPASSAAAGSGFIPANVLTSQILPVIKR.S] + [+15.9949 at M3]
Endoglucanase 1	79	49965	3	3	1112.7243 [K.SILFYEGQR.S];<br>1327.8185 [K.NTEEFQLYKR.H];<br>2189.1677 [K.MSYMVGFGDKYPQHVHHR.G]
Berberine bridge enzyme-like 2	43	41055	3	3	1180.5679 [K.TGLVFNPYGGR.M];<br>1180.5908 [K.TGLVFNPYGGR.M];<br>1353.8410 [R.ASFVSLFLGDAAR.L]
Phosphoenolpyruvate carboxylase, housekeeping isozyme	42	109753	3	3	1148.5394 [R.LATPELEYGR.M];<br>1878.9000 [K.NQTVDLVLTAHPTQSVR.R];<br>1992.9315 [K.VSEDDKLVEYDALLLDR.F]
Sucrose synthase 2	119	30453	4	4	1331.6473 [K.ESLEPLLDFLR.V];<br>1573.7646[R.NKESLEPLLDFLR.V];<br>2086.9428 [R.VNVHELSVDQLSVSEYLR.F];<br>2086.9709 [R.VNVHELSVDQLSVSEYLR.F]
UTP-glucose-1-phosphate uridylyltransferase	45	53619	2	2	1362.6585 [R.FFDNAIGINVPR.S];<br>1833.8451 [K.SNIEIHTFNQSQYPR.V]
Glyco_hydro_32C domain-containing protein	63	16679	1	1	1699.7890 [R.VLVDHSIVEGFSQGGR.S]
Epidermis-specific secreted glycoprotein EP1	55	55501	6	6	2284.1161 [K.YVNEGEFGPYIVEYDGNYR.T];<br>2284.1931 [K.YVNEGEFGPYIVEYDGNYR.T];<br>2284.1971 [K.YVNEGEFGPYIVEYDGNYR.T];<br>2284.2597 [K.YVNEGEFGPYIVEYDGNYR.T];<br>2284.423 [K.YVNEGEFGPYIVEYDGNYR.T];<br>2290.1955 [K.YVNEGEFGPYIVEYDGNYR.T] + [+6.0138 at V2]
Peroxidase	109	31797	3	3	1769.9244 [R.DSVVALGGPTWTLQLGR.R];

					1926.0381 [R.DSVVALGGPTWTLQLGRR.D]; 3293.5501 [K.QNICPSTGGDDNLSDLDETTTVFDNVYFR.N] + Carbamidomethyl (C)
Peroxidase 4	176	34039	3	3	1007.5200 [K.FQAQGLSTR.D]; 1499.7655 [R.DSVVILGGPDWDVK.L]; 2302.1545 [K.SQVESVCPGVVSCADIIAIAAR.D] + 2 Carbamidomethyl (C)
Acidic endochitinase	103	32331	5	5	1331.7860 [K.YSYVNIAFLNK.F]; 1512.7266 [K.YYDDQSGYSSSIK.S]; 3117.4317 [K.FGNGQTPEINLAGHCNPASNGCTSVSTGIR.N] + 2 Carbamidomethyl (C) + [+0.9970 at V25]; 3117.4720 [K.FGNGQTPEINLAGHCNPASNGCTSVSTGIR.N] + [+116.0110 at C22]; 3242.8219 [R.GFIPANVLTSQILPVIKR.S] + [+1278.3691 at T9]
Class IV chitinase	65	27511	3	3	1860.1154 [R.AINGAVECNGGNTAAVNAR.V] + [+58.0055 at C8]; 2435.9484 [R.AAFLSALNSYSGFGNDGSTDANKR.E] + [-27.0109 at N22]; 2463.2123 [R.AAFLSALNSYSGFGNDGSTDANKR.E]
Chitinase 5	103	28332	5	5	1542.7243 [R.AAFLSALNSYPGFGK.D]; 1542.8376 [R.AAFLSALNSYPGFGK.D]; 1792.7958 [R.GPLQISWNYNYGPAGR.S]; 1792.8889 [R.GPLQISWNYNYGPAGR.S]; 1808.8948 [R.GPLQISWNYNYGPAGR.S] + [+15.9772 at S6]
Annexin D2	45	38599	2	2	1351.7095 [R.AVLLWTPVPAER.D]; 1725.6804 [K.SLEEDVAYHTSGDFR.K]
Annexin D1	44	38599	2	2	1351.7095 [R.AVLLWTPVPAER.D]; 1725.6804 [K.SLEEDVAYHTSGDFR.K]
Glutaredoxin-dependent peroxiredoxin	110	19864	5	5	1189.6862 [R.RFALLVDDLK.V]; 1792.9078 [K.VIIFGVPGAFTPTCSVK.H] + Carbamidomethyl (C); 2021.9700 [K.VANVEAGGEFTVSSADDILK.A]; 2351.1393 [K.FLADGSATYTHALGLELDLSEK.G]; 2835.3856 [K.FLADGSATYTHALGLELDLSEKGLGTR.S]
Universal stress protein PHOS34	81	23014	1	1	1601.7685 [K.SVTDVILEVVEGDAR.N]
Retrotrans_gag domain-containing protein	26	198008	2	2	1555.8112 [K.ENEQPKIVNEDLK.R]; 1555.9097 [K.ENEQPKIVNEDLK.R]
Peptidyl-prolyl cis-trans isomerase	38	18301	3	3	1392.6745 [R.VFFDVSIGGAPAGR.I]; 1392.6793 [R.VFFDVSIGGAPAGR.I]; 1408.7124 [R.IVMELYADTTPR.T]
Cationic peroxidase 1	116	31881	5	2	1769.9088 [R.DSVVALGGPTWTLQLGR.R]; 2288.0736 [K.SQVESLCPGVVSCADIVAVAAR.D] +[2 Carbamidomethyl (C) +57.02 at C7,C13]; 2677.2003 [R.DSTTASLSTANSDLPGPASDLSTLISR.F]; 3276.1694 [K.ICPSTGGDNNLSDLDETTTVFDNVYFR.N]

					+ [PhosphoHexNAc (S) +283.0457 at S12]; 3292.1829 [K.QKICPSTGGDNNLSDLDETTTVFDNVYFR.N] + [Carbamyl (N-term) +43.0058 at N-term Q]
GDSL esterase/lipase	139	43198	6	6	1590.9352 [K.FTAAVQHLYQEGAR.T]; 1591.1930 [K.FTAAVQHLYQEGAR.T]; 1915.9670 [K.ALYTFDIGQNDLSVGFR.Q]; 1916.1042 [K.ALYTFDIGQNDLSVGFR.Q]; 1916.1584 [K.ALYTFDIGQNDLSVGFR.Q]; 1916.4225 [K.ALYTFDIGQNDLSVGFR.Q]
Endochitinase EP3	63	25100	2	2	2435.9484 [R.AAFLSALNSYSGFGNDGSTDANKR.E] + [-27.0109 at N22]; 2463.2123 [R.AAFLSALNSYSGFGNDGSTDANKR.E]
5-methyltetrahydropteroyltriglutamate--homocysteine S-methyltransferase	55	84627	7	7	1096.5282 [K.YLFAGVVDGR.N]; 1167.5502 [K.GVTGFGFDLVR.G]; 1469.7540 [K.AGITVIQIDEAALR.E]; 1658.7187 [K.YGAGIGPGVYDIHSPR.I]; 2086.9673 [K.LPVLPTTTIGSFPQTMDLR.R]; 2218.9747 [K.ALAGHKEEAFFSDNAVAQASR.K]; 2385.8709 [K.MLAVLETNILWVNPDCGLKTR.K]
Heat shock 70 kDa protein	94	72831	4	4	1675.6888 [K.ATAGDTHLGGEDFDNR.M]; 1680.7798 [K.NAVVTVPAYFNDSQR.Q]; 2658.1529 [K.EQVFSTYSDNQPGVLIQVYEGER.T]; 3025.3222 [R.TLSSTAQTTIEIDSLYEGIDFYSTITR.A]
Phosphopyruvate hydratase	202	46632	5	5	978.4605 [K.FRAPVEPY.-]; 978.5214 [K.FRAPVEPY.-]; 1877.8697 [R.GNPTVEVDVTLSDGTFAR.A]; 2252.0626 [R.SGETEDTFIADLSVGLATGQIK.T]; 2624.1222 [R.QIFDSRGNPTVEVDVTLSDGTFAR.A]
Fructose-bisphosphate aldolase	95	36262	4	4	1348.6057 [K.VSPEVVAEYTVR.T]; 1476.6755 [K.KVSPEVVAEYTVR.T]; 1570.7037 [R.LASINVENVEGNRR.A]; 2137.9409 [K.IGPTEPSELAIHENAYGLAR.Y]
Glyceraldehyde-3-phosphate dehydrogenase	129	36089	5	3	1731.7551 [K.VVAWYDNEWGYSSR.V]; 1745.7728 [K.VVAWYDNEWGYSSR.V] + [+14.0157 at D6]; 1748.7644 [K.VVAWYDNEWGYSSR.V] + [+17.0345 at D6]; 1993.0357 [K.DSNTLLFGEKPVTVFGIR.N]; 2185.9959 [K.GILGYTEDDVVSTDFIGDNR.S]
Isoflavone reductase-like protein	68	33980	3	3	1124.5836 [R.LGHPTFALIR.E]; 1429.5581 [R.FFPSEFGNDVDR.V]; 2067.9218 [K.SSGVSLVYGDLYDHESLVK.A]
Abscisic stress-ripening protein 2	126	23174	3	3	2186.0184 [K.HLEHLGELGVAAAGAYALHEK.H]; 2491.0821 [K.IEEEIAAAAAVGAGGFAFHEHHEK.K]; 2756.1925 [K.HKIEEEIAAAAAVGAGGFAFHEHHEK.K]
Usp domain-containing protein	81	17167	2	2	1601.7685 [K.SVTDVILEVVEGDAR.N]; 1601.7870 [K.SVTDVILEVVEGDAR.N]
Purple acid	162	68455	4	4	1346.6671 [R.FIEHCLASVDR.Q] +

phosphatase					Carbamidomethyl (C); 2086.1699 [K.LVAVSNSVAFANPNAPVYPR.L]; 2371.1169 [R.VLGYSSSSFYAEEGSFAEPMGR.D]; 2635.2032 [R.VVIFGDMGKDEADGSNEYNQYQR.G] + [-0.9840 at C-term R]
UDP-arabinopyranose mutase	91	40771	4	3	1487.7656 [R.VPDGFDYELYNR.N]; 1501.7844 [R.VPDGFDYELYNR.N] + [+13.9793 at P2]; 2122.1843 [R.ELIGPAMYFGLMGDGQPIGR.Y]; 2275.4445 [M.AGTPATPLLKDELDIVIPTIR.N] + [+42.0106 at T3]

Supplementary Table S3. List of phenolic compounds identified in *V. vinifera* callus-derived ENs by HPLC-UV-ESI-MS(MS^2)

No	Rt, min	Compound assignment	UV max, nm	Molecular Formula	ESI-MS(MS^2) data		
					$[M-H]^-$, *m/z*	Error, mDa	MS^2, *m/z*
1	29.9	*trans*-*δ*-Viniferin diglycoside I	311, 323	C40 H42 O16	777.2409	0.9	615, 453, 369
2	30.3	*trans*-*δ*-Viniferin diglycoside II	310, 322sh	C40 H42 O16	777.2412	1.2	615, 453
3	33.1	*trans*-*δ*-Viniferin glycoside I	310, 323	C34 H32 O11	615.1858	1.4	453, 435, 411, 369
4	33.8	*trans*-*δ*-Viniferin glycoside II	310, 320s	C34 H32 O11	615.1856	1.6	453, 435, 411, 369
5	35.2	*cis*-*δ*-Viniferin glycoside	284, 302sh	C34 H32 O11	615.1857	1.5	453, 435, 411, 369
6	38.5	*trans*-*δ*-Viniferin	310, 320sh	C28 H22 O6	453.1332	0.5	435, 411, 369, 347, 333

A

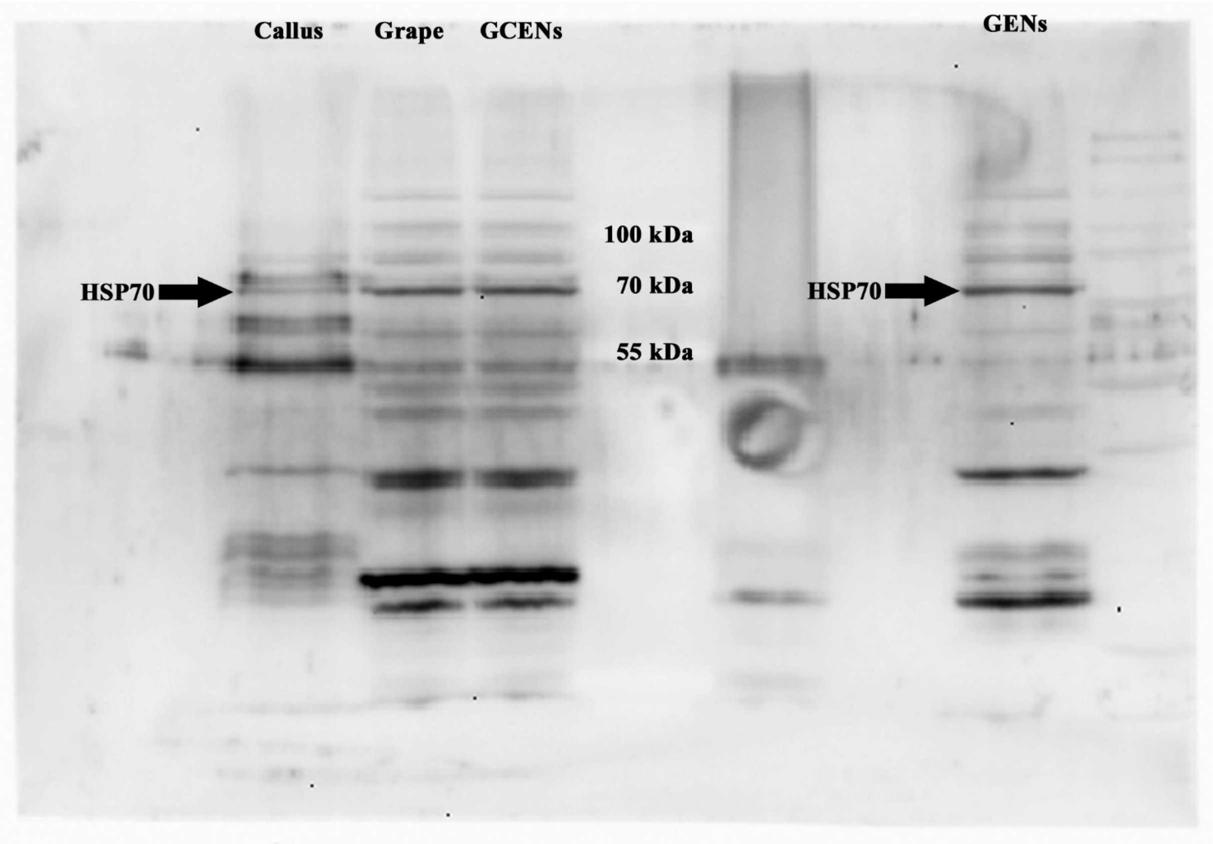

B

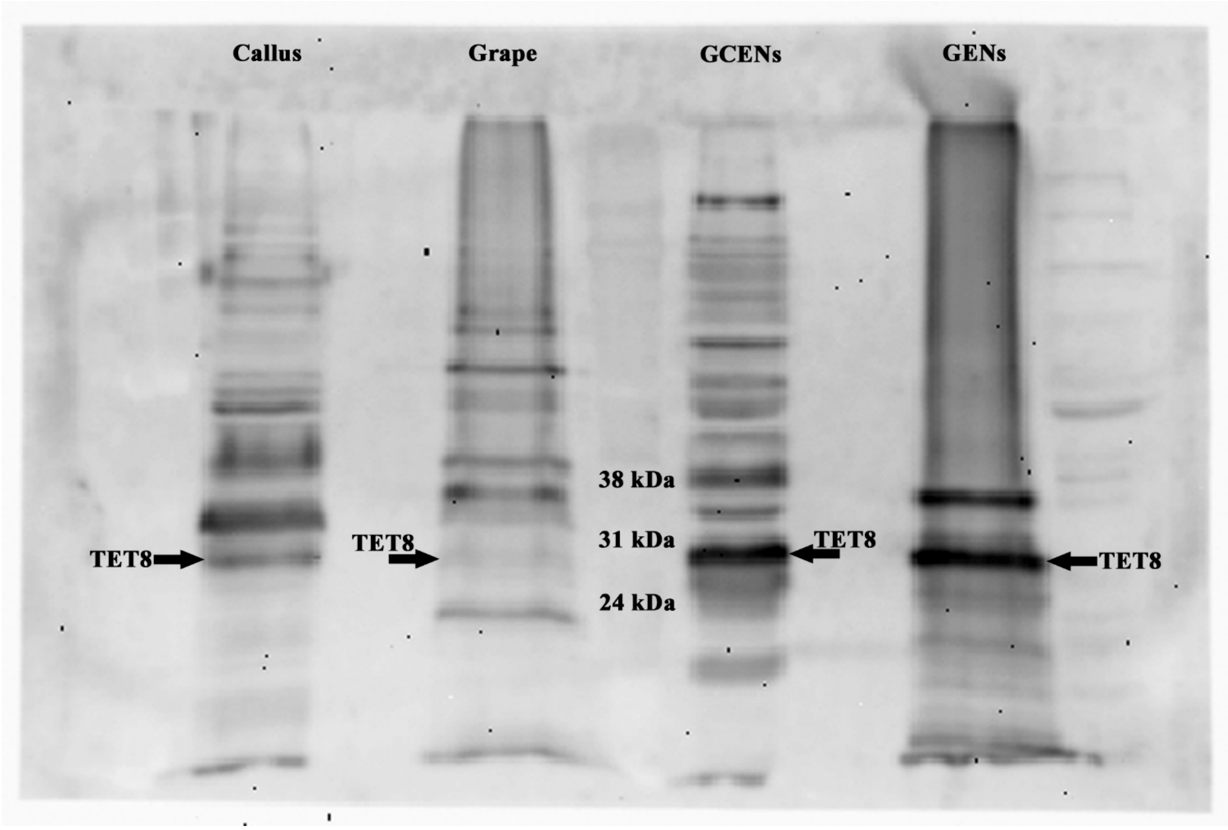

Supplementary Figure S1. Western blot analysis of ENs isolated from *V. vinifera* calli and berries juice. Proteins (40 μg) were resolved using a 10% SDS-PAGE gel. EVs marker proteins detected were: (**A**) HSP70 (71/72 kDa) and (**B**) TET8 (31 kDa).
